# Strategies to promote tendon-bone healing after anterior cruciate ligament reconstruction: Present and future

**DOI:** 10.3389/fbioe.2023.1104214

**Published:** 2023-03-13

**Authors:** Bin Tian, Ming Zhang, Xin Kang

**Affiliations:** Honghui Hospital, Xi’an Jiaotong University, Xi’an, China

**Keywords:** anterior cruciate ligament, tendon-bone healing, tendon-bone insertion, stem cells, cytokines, platelet-rich plasma, exosome

## Abstract

At present, anterior cruciate ligament (ACL) reconstruction still has a high failure rate. Tendon graft and bone tunnel surface angiogenesis and bony ingrowth are the main physiological processes of tendon-bone healing, and also the main reasons for the postoperative efficacy of ACL reconstruction. Poor tendon-bone healing has been also identified as one of the main causes of unsatisfactory treatment outcomes. The physiological process of tendon-bone healing is complicated because the tendon-bone junction requires the organic fusion of the tendon graft with the bone tissue. The failure of the operation is often caused by tendon dislocation or scar healing. Therefore, it is important to study the possible risk factors for tendon-bone healing and strategies to promote it. This review comprehensively analyzed the risk factors contributing to tendon-bone healing failure after ACL reconstruction. Additionally, we discuss the current strategies used to promote tendon-bone healing following ACL reconstruction.

## 1 Introduction

**FIGURE 1:**
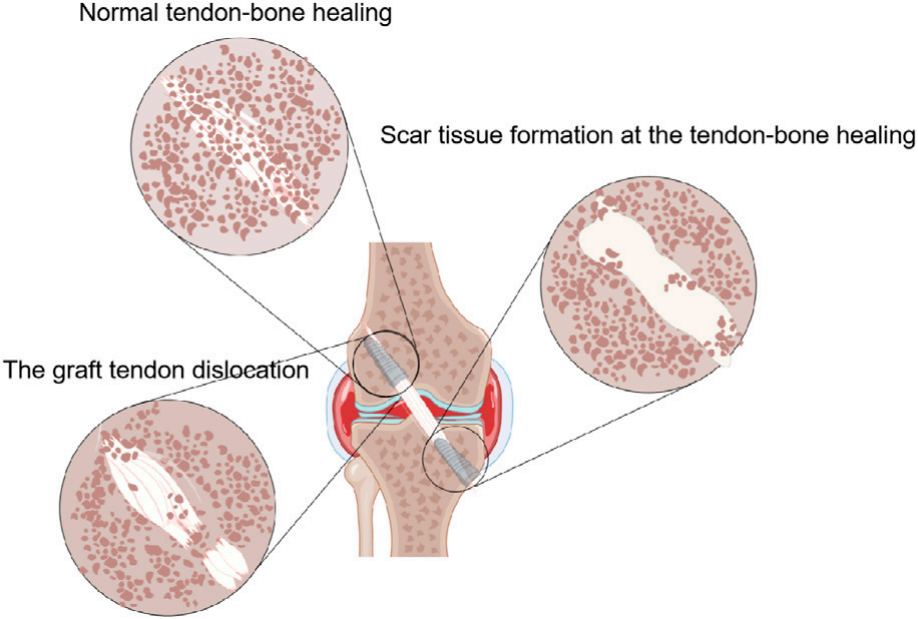
The outcome of tendon-bone healing is presented by Figdraw. In normal tendon-bone healing, the transplanted tendon will eventually be fully ossified in the bone tunnel. Scar tissue formation at the tendon-bone healing interface will result in poor mechanical properties. Secondly, failure of graft tendon fusion in the bone tunnel will lead to graft dislocation.

In recent years, with the extensive development of sports, the incidence of Anterior cruciate ligament (ACL) injury increases year by year (Best et al., 2021). At present, arthroscopic ACL reconstruction is the main treatment (Majumdar et al., 2022). It can rapidly restore knee function and reduce the incidence of osteoarthritis (Mather et al., 2013). Tendon -bone healing is the key factor determining the outcome of ACL reconstruction. The physiological process of tendon-bone healing is complicated because the tendon-bone junction is a transition from ligaments to bone tissue (Lu et al., 2019; Zhao et al., 2022). The transplanted tendon must be passed through a bone tunnel during ACL reconstruction, which makes tendon healing with bone tissue challenging because the process requires the fusion of two different tissues. In normal tendon-bone healing, the transplanted tendon will eventually be fully ossified in the bone tunnel (Zhao et al., 2022). Scar tissue formation at the tendon-bone healing interface will result in poor mechanical properties. Secondly, failure of graft tendon fusion in the bone tunnel will lead to graft dislocation, which will eventually impair the stability of the knee joint (Figure 1).

Perfect tendon-bone healing is crucial for ACL reconstruction, so it is important to study the risk factors that may affect tendon-bone healing. After ACL reconstruction, the patient’s bone tunnel needs to undergo four stages of inflammation, hyperplasia, remodeling and maturation. This process is influenced by many factors, including age, the way graft tendon is fixed, the mechanical load of graft tendon and the condition of the bone marrow canal (Hsu and Wang, 2013; Packer et al., 2014; Uefuji et al., 2014; Camp et al., 2017; Zhao et al., 2019; Tei et al., 2020). Therefore, in order to avoid the influence of these factors on tendon transplantation, a variety of clinical measures should be taken, including the use of magnesium based interference screw to fix the transplanted tendon, moderate postoperative rehabilitation training, etc. (Ammann et al., 2022). Therefore, only attention to the above influencing factors can ensure the perfect fusion of the transplanted tendon in the bone tunnel, so as to enable patients to recover the knee function earlier and faster (Woo et al., 2004).

Furthermore, researchers have adopted a variety of strategies to promote healing of transplanted tendon and bone tissue. It mainly includes biomaterials, stem cells, cytokines and mechanical stimulation strategies. Researchers also have conducted a large number of basic studies on the above strategies in the animal model of ACL reconstruction, and achieved certain results in the mechanism of action and functional efficacy methods. A number of preclinical studies were conducted involving the above strategies individually or in combination, which laid a theoretical foundation for the optimization of strategies to promote tendon-bone healing. Some of these strategies have also been studied clinically and have achieved initial efficacy.

This review systematically analyzed the risk factors that may affect ACL reconstruction and their coping strategies. Secondly, it comprehensively summarized the strategies to promote tendon-bone healing after ACL reconstruction and analyzed the potential value, providing a theoretical basis for the treatment of tendon-bone healing after ACL reconstruction.

## 2 Factors that may influence tendon-bone healing of the ACL reconstruction

**FIGURE 2:**
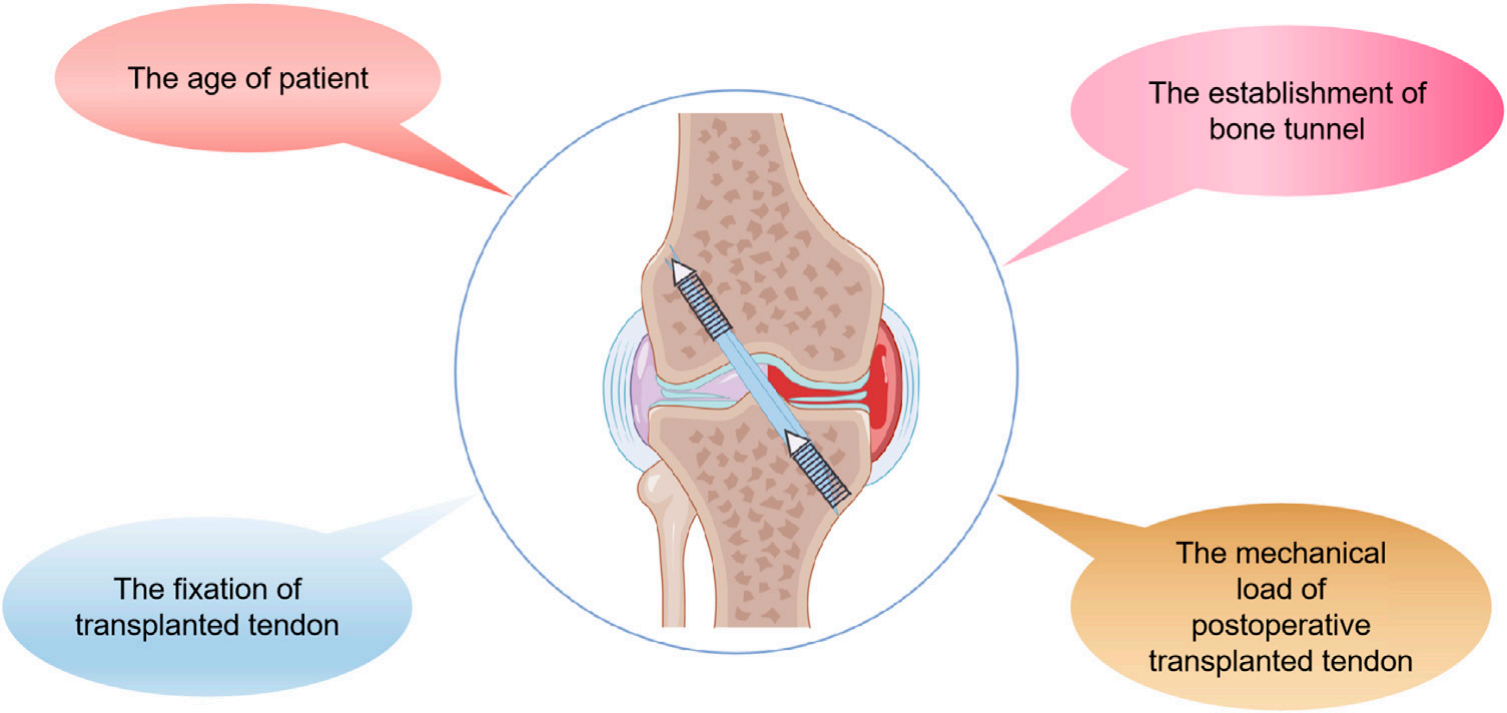
The main factors that may affect tendon-bone healing after ACL reconstruction are presented by Figdraw, including the age of patient, the fixation of the transplanted tendon, the establishment of the bone tunnel and the mechanical load of the postoperative transplanted tendon.

There are many variables that influence tendon-bone healing after ACL reconstruction. In recent years, studies have confirmed that the main factors affecting tendon-bone healing include the age of patient, the fixation of transplanted tendon, the establishment of bone tunnel and the mechanical load of postoperative transplanted tendon (Figure 2) (Table 1). In addition, the effect of Non-Steroidal Antiinflammatory Drugs (NSAIDS) on tendon-bone healing is still controversial (Duchman et al., 2019). NSAIDS should be used with caution in order to ensure adequate pain relief while limiting unnecessary exposure to unknown side effects (Soreide et al., 2016). Thus, it can better promote postoperative tendon-bone healing and enable patients to return to physical activity earlier, faster and more safely.

**TABLE 1:** Factors that may influence tendon-bone healing after ACL reconstruction.

Influence factors	Mechanism	Animal model	References
Age of patient	With age, CD34+ stem/progenitor cells are also reduced in ACL tissue, and thus the ability of tendon - bone healing after ACL reconstruction is correspondingly weakened.	Patients	Uefuji et al. (2014)
Age of patient	The younger patients had better tendon-bone healing and biomechanical characteristics.	Patients	Magnitskaya et al. (2020)
Age of patient	The age was a predictor of 2-year regression exercise, and the younger the age, the more likely it was to return to exercise 2 years after surgery.	Patients	Faleide et al. (2021)
Fixation of the transplanted tendon	Different tendon fixation methods may affect the efficacy of tendon-bone healing after ACL reconstruction.	Rabbits	Hsu and Wang (2013)
The shape of the bone tunnel	Biomechanical experiments have also shown that ACL reconstruction using a flat bone tunnel leads to stronger tendon-bone healing than a round bone tunnel.	Rabbits	Zhao et al. (2019)
Thermal damage caused by motorized drilling	Thermal necrosis caused by motorized drilling in bone tunnels may lead to delayed tendon-bone healing. Manual drilling preserves the bone stock in the tunnel, reduces thermal necrosis, and provides a better microenvironment for rapid healing of the interface.	Rabbits	Tei et al. (2020)
The mechanical load of the transplanted tendon	High mechanical loads applied immediately after surgery appear to have adverse effects on healing in rat models. Delayed mechanical loading is beneficial to the healing of the tendon-bone interface. Avoiding high-tension movement of the graft early after surgery may improve tendon-to-bone healing in humans.	Rats	Packer et al. (2014)
The mechanical load of the transplanted tendon	It has been shown that a short period of time without mechanical loading of the transplanted tendon after ACL reconstruction seems to improve the biomechanical strength of the healed tendon-bone interface, while a long period without mechanical loading may lead to a decrease in the ultimate load.	Mice	Camp et al. (2017)

### 2.1 Age factors

Clinically, it was found that patients of different ages had different responses to treatment. Some researchers have explored the relationship between age and the healing potential of the ACL. CD34+ stem/progenitor cells from ACL remnants contribute to tendon-bone healing. Uefuji et al. (2014) collected ACL remnants from 28 patients with arthroscopic ACL reconstruction. The CD34+ stem/progenitor cell characteristics of ACL remnant tissue were analyzed. The results showed that the number of CD34+ cells in adolescent ACL residues was significantly higher than that in patients over 30 years old, and had higher potential for proliferation and multiline differentiation *in vitro*. This study showed that CD34+ stem/progenitor cells decreased in ACL tissue with increasing age, and the ability of tendon-bone healing after ACL reconstruction was correspondingly weakened. This study may provide a theoretical basis for explaining that aging may be a risk factor for tendon-bone healing failure.

A growing number of clinical studies have confirmed the above theory in recent years. Magnitskaya et al. (2020) conducted a retrospective case-control study on 298 patients who received ACL reconstruction surgery. The results show that the main individual characteristic affecting International Knee Documentation Committee (IKDC) 2000 score is age. The IKDC 2000 score of patients under 30 years old was higher than that of control group at all time points. This study also reflects laterally that the younger patients may obtain the better tendon-bone healing results after ACL reconstruction. Similarly, Taylor et al. (2015) made a systematic analysis of clinical studies on the primary repair of the ACL. It was also found that younger patients had better tendon-bone healing and biomechanical characteristics. In addition, Faleide et al. (2021) explored the predictive indicators of regression movement in patients 2 years after ACL reconstruction. The results also found that age was a predictor of 2-year regression exercise, and the younger the age, the more likely it was to return to exercise 2 years after surgery. All these studies indicate that age factors influence the efficacy of tendon-bone healing after ACL reconstruction. Therefore, more efforts may be needed to promote tendon-bone healing in older patients in the clinic.

### 2.2 Fixation of the transplanted tendon

During ACL reconstruction, the fixation method of the tendon may also effect tendon-bone healing postoperatively. In the process of ACL reconstruction, in order to obtain proper tension, it is particularly important to select an appropriate fixation method to fix the transplanted tendon in the bone tunnel. At present, the method of external tunnel fixation generally uses the combination of button, gasket and scaffold to suspend the graft tendon on the surface of adjacent cortical bone outside the tunnel. Metal or bioabsorbable interference screws are used for internal fixation in tunnels (Chen et al., 2007). There is no consensus on the optimal fixation method for tendon transplantation in ACL reconstruction. Nevertheless, different fixation methods have both advantages and disadvantages.

Suspension fixation maximizes the amount of bone graft in the bone tunnel, thereby improving the effectiveness of ACL reconstruction. This technique avoids common problems that interfere with screw fixation, such as screw placement deviation, thread cutting of the graft tendon, and increased difficulty in revision surgery in the presence of screws (Barrett et al., 2002). However, the initial strength and tolerable mechanical load of suspension fixation are low. Thus, metal interference screws are still somewhat of the gold standard, with their high initial fixed strength and mechanical load. Metal interference screw has good clinical effect and low complication rate. As a downside, it interferes with magnetic resonance imaging (MRI) and has to be removed during revision surgery (Emond et al., 2011). With the development of biomaterials, bioabsorbable interference screws have been introduced to the clinic, with some patients preferring the clinical outcome of the screw disappearing (Xu et al., 2021). Most current metal interference screws are composed of titanium, but bioabsorbable interference screws are composed of a variety of biodegradable biomaterials that are non-toxic, non-responsive to tissue, and degrade over time (Xu et al., 2021). However, there have been some clinical reports of adverse reactions related to bioabsorbable interference screws, such as the risk of synovitis, excessive inflammation, and tunnel widening (Xu et al., 2021). In a study comparing bioabsorptive interference screws with metal ones, Debieux et al. (2016) demonstrated no differences in patients’ self-reported knee function or postoperative activity. Therefore, the above indicated that different ways of tendon transplantation fixation may have different prognostic results, and may also affect postoperative tendon-bone healing. Appropriate tendon fixation measures during ACL reconstruction play an important role in postoperative recovery of patients.

### 2.3 Establishment of bone tunnel

Recently, the method of reconstructing the ACL has changed greatly with the enhanced understanding of its anatomy. Some researchers have changed the shape of the bone tunnel to make it more closely match the tendon and insertion position of the ACL. These methods include ACL reconstruction of elliptical, rectangular and flat bone tunnels. Biomechanical studies on cadavers suggest that flat tunnel ACL reconstruction may result in better knee stability than traditional round tunnel ACL reconstruction (Nakase et al., 2016; Fink et al., 2018; Hiramatsu et al., 2018; Tachibana et al., 2018; Mae et al., 2019). Zhao et al. (2019) evaluated whether flat bone tunnel has a positive impact on the early tendon-bone healing process after ACL reconstruction. In the rabbit ACL reconstruction model, it was found that the cell and collagen remodeling process was faster in the flat tunnel group, more fibrochondrogenesis, and more new bone formation in the bone interface area. This study showed that early flat bone tunnel accelerated tendon-bone healing after ACL reconstruction in rabbits, which laid a foundation for further clinical application of this method.

In addition, studies in animal models have shown that mechano-thermal stress during the establishment of bone marrow canals can delay bone healing and increase bone resorption, leading to the possibility of graft integration failure (Eriksson et al., 1984; Packer et al., 2014; Tei et al., 2020). Thermal necrosis caused by electric drilling during ACL reconstruction may be a factor affecting delayed healing of the tendon-bone interface. Tei et al. (2020) compared the effects of artificial drilling and standard electric drilling on the tendon-bone interface after ACL reconstruction from the perspectives of histology and biomechanics. Studies have demonstrated that manual drilling preserves bone stock in bone tunnels, reduces thermal necrosis, and provides a better microenvironment for rapid healing of the interface. However, electric drilling results in thermal necrosis of bone cells and extensive bone loss in bone tunnels, resulting in enlarged bone tunnels and delayed tendon-bone healing. The use of manual drilling to establish bone tunnels during ACL reconstruction can preserve tissue viability by reducing osteonecrosis, potentially benefiting patients in the clinic.

In conclusion, the shape and method of bone tunnel construction during ACL reconstruction may affect postoperative tendon-bone healing. Therefore, the use of better tunnel shape and suitable drilling equipment in ACL reconstruction may be of great significance in promoting tendon-bone healing after surgery.

### 2.4 Mechanical load of postoperative tendon transplantation

Tendon-bone interfaces are characterized by unique mechanical stress environments due to the unique organization of the area and the presence of two materials with contrasting mechanical properties (Yang and Temenoff, 2009; Font Tellado et al., 2015). Additionally, the formation of tendon-bone structure is also influenced by mechanical loads (Font Tellado et al., 2015; Carbone et al., 2016). Mesenchymal stem cells (MSCs) are considered to be key factors involved in the repair of tendon-bone injury because they infiltrate the surrounding tendon graft to participate in tendon-bone healing through the bony tunnel wall established during ACL reconstruction (Kida et al., 2013). The use of MSCs to accelerate early tendon-bone healing has been the subject of a large number of studies in recent years (Rizzello et al., 2012; Gao et al., 2016). Studies have shown that MSCs can enhance proliferation, differentiation and inhibit apoptosis under mechanical stimulation (Chen and Jacobs, 2013; Bin et al., 2016). Therefore, by stimulating MSCs regeneration, mechanical loading may enhance tendon-bone healing.

After ACL reconstruction, passive exercise therapy has been investigated in numerous studies. Using rabbit ACL reconstruction model, Song et al. (2017) studied the effects of mechanical loading on tendon-bone healing. The results showed that passive exercise therapy significantly promoted tendon-bone healing, which was manifested as increased number of fibrocartilage and upregulated expression of collagen Ⅰ, alkaline phosphatase and osteopontin genes at the tendon-bone junction. In this study, mechanical stimulation was found to enhance MSCs regenerative potential in tendon-bone healing. Additionally, researchers examined how mechanical load starts to be exerted on tendon grafts in bone tunnels and how that affects healing. It was found by Camp et al. (2017) that mice with passive knee movements from 5 days after surgery had improved tendon-bone healing. This study have demonstrated that a period of non-weight bearing early after surgery appears to improve the biomechanical strength of the tendon-bone interface, while prolonged absence of mechanical load results in a reduction of ultimate load. Besides, Packer et al. (2014) demonstrated that loading mechanical load immediately after surgery can intensify tendon-bone healing by delaying the application of low-strength strain mechanical load compared with long-term fixation. The application of high-intensity mechanical loading immediately after surgery appears to have adverse effects on the healing of rat models. These studies suggest that the knee is not easily subjected to immediate mechanical loading after surgery, and delayed application of low-intensity mechanical loading can improve tendon-bone healing. It also provides a theoretical basis for how to restore functional exercise after surgery. The researchers have also supported this theory through clinical studies. Isberg et al. (2006) evaluated the effect of early functional exercise on knee slack after ACL reconstruction in a prospective randomized controlled study, which found that early functional exercise did not increase knee slack 2 years after ACL reconstruction. In addition, Ebert et al. (2022) confirmed in a randomized controlled trial that early functional exercise after ACL reconstruction can improve knee joint strength without increasing graft relaxation.

## 3 Preclinical study of strategies to promote tendon-bone healing in ACL reconstruction

**FIGURE 3:**
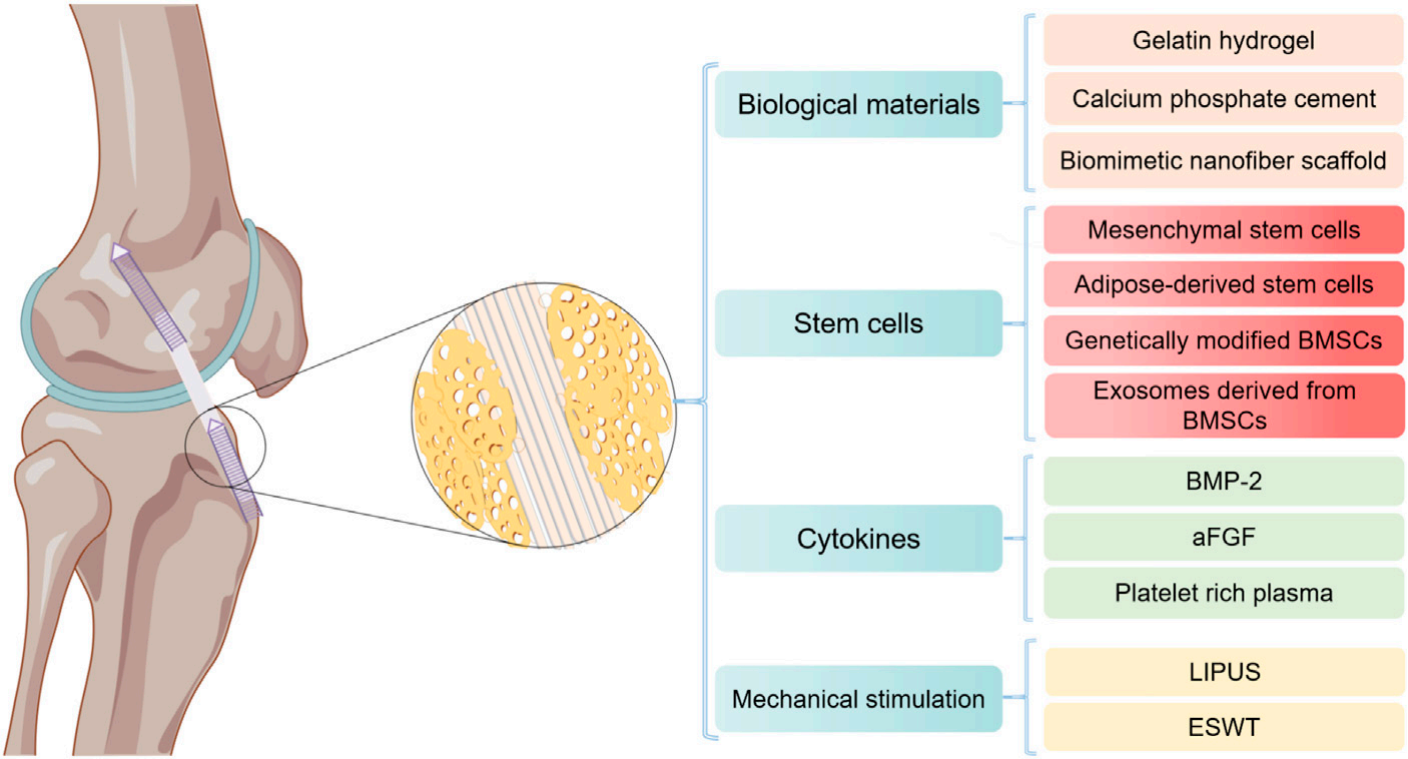
Major strategies for promoting tendon-bone healing after ACL reconstruction by Figdraw. BMSCs, bone mesenchymal stem cells; BMP-2, bone morphogenetic protein-2; aFGF, acidic fibroblast growth factor; LIPUS, low intensity pulsed ultrasound; ESW, extracorporeal shock wave.

Although autologous tendon transplantation for ACL reconstruction has made great progress, there are still limitations and challenges. Autograft therapy has the disadvantage of a longer healing time for tendon-bone connections and unpredictability of results (Goradia et al., 2000). More importantly, true bone tunnel healing is driven by the formation of fibrous scar tissue, whose structure and composition are inferior to that of primary tissue (Freedman et al., 2017). It is inevitable that the bone tunnel will be subjected to high mechanical stress after ACL reconstruction, which may lead to failure of the reconstruction (Smith et al., 2012; Lu and Thomopoulos, 2013). Consequently, tendon-bone healing needs to be strengthened and accelerated. Restoring the original structure and function of the tendon-bone interface is the ideal method of tendon-bone healing. The final bone tunnel is composed of ligament, fibrocartilage, calcified fibrocartilage and bone (Schwartz et al., 2012; Deymier-Black et al., 2015), which are mainly composed of type I collagen fibers, mineral components and a small amount of proteoglycan (Smith et al., 2012). In recent years, studies have adopted biological materials, stem cell transplantation, cytokines, mechanical stimulation and other intervention means, in order to achieve a better effect of promoting tendon-bone healing in autograft therapy (Weimin et al., 2013; Patel et al., 2018) (Figure 3). In addition, nano-biomimetic scaffolds have been used to replace autologous tendon for ACL reconstruction, and preliminary results have been obtained (Cai et al., 2018). However, most of these techniques are limited to preclinical animal model studies and have not been widely used in clinical practice.

### 3.1 Biological materials

In the past decade, strontium-enriched calcium phosphate cement (Sr-CPC), hydrogel microspheres, nano-biomimetic scaffolds, nanofiber membrane, hydroxyapatite (HA) and other inorganic bone components have been widely used in preclinical studies to promote tendon-bone healing after ACL reconstruction due to their advantages in histocompatibility and osteogenic induction (Table 2). Studies have been conducted on promoting tendon-bone healing with the use of the above materials alone or in combination with stem cells or cytokines, and certain achievements have been made. Clinical trials are expected to demonstrate good efficacy in the near future.

**TABLE 2:** Preclinical studies of biomaterials promoting tendon-bone healing in ACL reconstruction.

Intervening measure	Animal model	Efficacy in promoting tendon-bone healing	References
Strontium-enriched calcium phosphate cement (Sr-CPC)	Rabbit	Sr-CPC promotes osteoblast-like cell proliferation.	Kuang et al. (2013)
Simvastatin coupled gelatin hydrogel	Rabbit	Simvastatin coupled gelatin hydrogel promotes early tendon-bone healing through angiogenesis and osteogenesis.	Oka et al. (2013)
Strontium-enriched calcium phosphate cement (Sr-CPC)	Rabbit	Sr-CPC promotes healing of the transplanted tendon in the bone tunnel.	Kuang et al. (2014)
Biomimetic nanofiber membrane of polycaprolactone/nanohydroxyapatite/collagen (PCL/nHAp/Col)	Rabbit	PCL/nHAp/Col nanofiber membrane can effectively promote the healing of tendon and host bone and improve the mechanical strength.	Han et al. (2015)
Biodegradable polylactide (PLA) bolt as the bone anchor and apoly (d,l-lactide-co-glycolide) (PLGA) nanofibrous membrane	Rabbit	PLGA nanofiber membrane can effectively reduce tunnel enlargement and enhance tendon-bone fusion.	Chou et al. (2016)
Polycaprolactone (PCL) electrospinning membrane and chitosan/hyaluronic acid (CS/HA) multilayer membrane formed the biomimetic nanofiber tendon bone fusion membrane	Rabbit	PCL-CS/HA multilayer nanofiber membrane can promote osteoblast proliferation and recruitment by loading cytokines. Wrapping the autogenous tendon with this membrane can provide better tendon-bone fusion and inhibit scar tissue formation.	Han et al. (2019a)
bFGF-loaded electrospun poly (PLGA) fibrous membrane	Rat	bFGF-PLGA membrane facilitates cell attachment and proliferation, and accelerates tendon-bone reconstruction.	Zhao et al. (2014)
Chitosan/gelatin/β-glycerol phosphate (C/G/GP) hydrogels	Rabbit	Collagenase C/G/GP hydrogel promotes tendon-bone healing.	Huang et al. (2020
Silk-collagen scaffold with both ends modified by hydroxyapatite	Rabbit	The silk-collagen scaffold can promote bone fusion at the tendon-bone interface.	Bi et al. (2021)
Dual-layer aligned-random scaffold (ARS)	Rabbit	By inducing new bone formation, ARS contributes to improved gradient microstructure by enhancing tendon-bone fusion.	Cai et al. (2018)

*In vitro* experiments showed that strontium can promote the proliferation of preosteoblast cells and the synthesis of bone matrix (Canalis et al., 1996). It has also been found to reduce osteoclast activity (Bonnelye et al., 2008). In recent years, Sr-CPC has been developed and tried to be applied in the study of promoting tendon-bone healing. Kuang et al. (2013) studied whether the transplanted tendon treated with Sr-CPC could accelerate the healing in bone tunnels during ACL reconstruction. Early bone formation in the initial space between the graft and host bone and subsequent accelerated healing of allograft soft tissue tendons in the bone tunnel were observed in a rabbit ACL reconstruction model using Sr-CPC preconditioning. Similarly, Kuang et al. (2014) also demonstrated that local application of Sr-CPC could accelerate graft healing in bone tunnels in an ACL reconstruction model. These studies suggest that Sr-CPC can enhance tendon-bone healing and thus improve the clinical outcome of ACL reconstruction.

As an effective drug carrier, hydrogel microspheres are widely used in the clinical research of drug delivery due to their high potency and delayed drug release. Oka et al. (2013) studied the efficacy of simvastatin coupled gelatin hydrogel implanted in bone tunnel to promote tendon-bone healing after ACL reconstruction. Simvastatin coupled gelatin hydrogel was implanted into the bone tunnel of rabbits during ACL reconstruction to observe the bone regeneration, neovasculation and biomechanical properties at the tendon-bone interface. It was found that local application of low-dose simvastatin combined with gelatin hydrogel promoted early tendon-bone healing through angiogenesis and osteogenesis. In addition, Huang et al. (2020) studied adding gelatin molecules into the thermosensitive chitosan/β-disodium glycerophosphate-saline gel to form chitosan/gelatin/β-glycerophosphate-C/G/GP hydrogel, which was applied to the tibial plateau bone tunnel of rabbits. Hydrogel binding of collagenase C/G/GP induces more braided bone formation at an early stage to promote tendon-bone healing. These studies indicate that hydrogels have great potential as an ideal drug carrier to promote tendon-bone healing.

Biomimetic micro/nano scale scaffolds with high porosity and high surface area can be prepared by electrospinning technology (Kim et al., 2015). Biomimetic nanoscaffolds have been extensively studied in the regeneration of nerve, bone and other tissues. Biomimetic nanoscaffolds have been applied in bone tissue repair for their good biocompatibility and properties of promoting bone cell adhesion and growth. Researchers are also trying to use it to improve tendon-bone healing. Bi et al. (2021) studied the effect of HA-modified sericin scaffold on promoting tendon-bone interface healing and preventing osteoarthritis in rabbits. The HA/sericinogen scaffold was prepared using degummed knitted silk scaffold, type I collagen matrix and simulated body fluid (SBF). It was also found to promote tendon-bone fusion after ACL reconstruction. A number of animal studies have shown HA/serricinogen scaffold to be helpful in promoting osseointegration. These studies indicate that nano-biomimetic fiber scaffold can enhance tendon-bone healing by increasing tissue invasion and cell attachment, which has great potential for clinical application. Furthermore, with the electrospinning method, Cai et al. (2018) prepared a double-layer poly nanofibrous scaffold made of silk fibroin fibers, and evaluated the scaffold’s effect on tenson-bone healing *in vivo*. The results showed that double-layer fiber scaffolds could effectively enhance the fusion of tendon-bone in rabbit extrarticular model and improve the gradient microstructure by inducing new bone formation of rabbit extrarticular bone. The results of these studies suggest that tissue engineering scaffolds can be used effectively to promote tendon-bone healing, and that the structure of tissue engineering scaffolds can be optimized to improve their function.

Han et al. (2015) prepared a bionic polycaprolactone/nano-hydroxyapatite/collagen nanofiber membrane (PCL/nHAp/Col), which simulated the composition of primary bone tissue. This membrane has excellent cytocompatibility, allowing osteoblasts to adhere, grow and form bone. It was found that PCL/nHAp/col wrapped tendons may provide better tissue integration than unwrapped tendons and improve the mechanical strength of the healing interface in rabbit ACL reconstruction models. Similarly, Chou et al. (2016) explored the role of collagen-embedded poly (d, L-lactate-co-Glycolide) nanofiber membrane (PLGA) in promoting tendon-bone interface integration. PLGA nanofibers were found to reduce bone tunnel enlargement and enhance tendon-bone fusion in rabbits. In addition, multilayer nanofiber membrane structures were prepared to load cytokines to promote tendon-bone healing. Han et al. (2019a) used polycaprolactone (PCL) electrospinning membrane and chitosan/hyaluronic acid (CS/HA) multilayer membrane to prepare biomimetic nanofiber membrane. The PCL nanofibers were loaded with stromal cell derived factor-1α and bone morphogenetic protein-2 (BMP-2). The membrane can promote cell proliferation and recruitment, and induce osteogenic differentiation and recruitment of bone mesenchymal stem cells (BMSCs). It was found that in rabbit ACL reconstruction model, the membrane wrapped autograft tendon can provide better tendon-bone fusion and inhibit scar tissue formation. More importantly, the biomechanical properties of the tendon-bone interface were also significantly improved. Due to the synergistic effect of dual growth factors released by the membrane, tendon-bone healing can be enhanced and fibrous scar tissue can be inhibited at the interface. Similarly, Zhao et al. (2014) invented an electrospun polylact-glycol ester (PLGE) fiber membrane loaded with bFGF for promoting tendon-bone healing. The results demonstrated that Electrospinning fiber membranes facilitated cell attachment and proliferation, as well as sineoskeletal reconstruction. The PLGE fiber membrane loaded with bFGF had a more pronounced effect on sineoskeletal healing. These studies indicate that biomimetic nanofiber membranes loaded with multiple cytokines have certain clinical application potential in promoting tendon-bone healing.

Recently, Jiang et al. (2023) developed an injectable hydroxyapatite/Type I collagen (HAp/Col I) paste to construct a suitable local microenvironment and to explore its role in promoting tendon-bone healing. In the study of canine ACL reconstruction, it was found that injectable HAp/Col I pastes can improve the tendone-bone healing of the ACL after autologous tendon reconstruction, as evidenced by medical imaging, biomechanical, and histological results. Injectable hydroxyapatite/type I collagen paste can be applied to promote sinew/bone healing after ACL reconstruction, which will be of great attraction to ligament regeneration and has great potential for clinical application.

### 3.2 Stem cells

MSCs are considered to be a key factor in promoting tendon-bone healing, because different sources of MSCs at the tendon-bone interface are involved in fibrochondral and bone formation during tendon-bone healing. MSCs have been successfully tested in a number of studies in recent years to promote early tendon-bone healing (Rizzello et al., 2012; Gao et al., 2016) (Table 3). With the mechanism of MSCs promoting tendon-bone healing gradually becoming clear, cytokine gene modified MSCs and MSCs-derived exosomes have gradually become the focus of research in this field in recent years, which is expected to make a breakthrough in promoting tendon-bone healing.

**TABLE 3:** Preclinical studies of stem cells in promoting tendon-bone healing in ACL reconstruction.

Intervening measure	Animal model	Efficacy in promoting tendon-bone healing	References
BMSCs genetically modified with BMP2 and bFGF	Rabbit	Gene modified MSCs can promote new bone formation and higher mechanical properties, and contribute to the healing process.	Chen et al. (2016)
Adipose-derived regenerative cell (ADRC)	Rabbit	ADRCs can enhance graft healing during ACL reconstruction.	Kosaka et al. (2016)
PRP combined with BMSCs	Rabbit	The combination of PRP and BMSCs promote more maturation of the tendon-bone interface, and more newly formed bone was found in the bone tunnel.	Teng et al, (2016)
Stromal cell-derived factor 1 (SDF-1)-releasing collagen-silk (CSF) scaffold combined with intra-articular injection of LSPCs	Rabbit	SDF-1-CSF scaffold combined with intraarticular injection of LSPCs can improve cartilage degeneration and reduced the severity of joint fibrosis.	Hu et al. (2018)
hBMSC	Rat	hBMSC can accelerate tendon-bone healing and enhances the proliferation, differentiation, and collagen synthesis of fibroblasts.	Sun et al. (2019)
ADSCs sheets	Rabbit	ADSCs sheets can prevent bone tunnel enlargement, and may contribute to early tendon-bone healing.	Matsumoto et al. (2021)
BMSCs modifcated of RUNX1 with lentiviral system	Rat	Runx1 overexpressed BMSCs can promote osteogenesis by upregating the number of osteoblasts at the tendon-bone interface.	Kang et al. (2022)
Exosome derived from magnetically actuated iron oxide nanoparticles (IONPs)	Rat	IONP-Exos can promote more bone inward growth in the tendon graft, and increase fibrochondrogenesis at the tendon-bone tunnel interface.	Wu et al. (2022a)
BMSC-Exos	Rat	BMSC-Exos can promote the polarization of M1 macrophages to M2 macrophages, and promote tendon-bone healing.	Li et al. (2022)
Exosomes from infrapatellar fat pad (IPFP) MSCs	Rat	IPFP MSC-derived exosomes accelerate intraarticular graft reconstruction after ACLR.	Xu et al. (2022a)

Sun et al. (2019) explored the effect of human bone marrow stem cells (hBMSC) on graft-bone fusion in a rat model of ACL reconstruction. Based on the results of this study, hBMSC promotes tendon-bone healing by enhancing fibroblast proliferation, differentiation, and collagen synthesis. Similarly, Kosaka et al. (2016) observed the effects of adipose-derived regenerative cells (ADRCs) on tendon-bone healing in a rabbit model. It was also observed that ADRCs-treated tissues had Sharpey-like fibers connecting transplanted tendons to bone tissue earlier, and the size and shape of chondroid cells at the tendon-bone healing interface was more regular and ordered. ADRCs were administered locally to promote early healing of tendon-bone junctions in this study. In addition, Matsumoto et al. (2021) also used adipose-derived stem cells (ADSCs) sheets in the study of ACL reconstruction and confirmed that ADSCs sheets could improve the biomechanical strength of the tendon-bone interface in the rabbit model, prevent the expansion of bone tunnel and promote the healing of the tendon-bone interface. These studies indicate that MSCs have great potential as a cell therapy technique to promote tendon-bone healing.

The use of stem cells transplantation in combination with other therapeutic approaches is also one direction to promote innovation in tendon-bone healing therapy. Teng et al. (2016) explored the ability of platelet-rich plasma (PRP) combined with BMSCs to promote tendon-bone healing in rabbit models. It was found that PRP can significantly stimulate BMSCs’ osteogenic differentiation and induce BMSCs’ osteogenic differentiation. The combination of PRP and BMSCs can promote tendon-bone healing in rabbit model of ACL reconstruction, showing its clinical application potential. In addition, Hu et al. (2018) studied the synergistic therapeutic effect of stromal cell derived factor 1 (SDF-1) releasing collagen filament (CSF) scaffold combined with intraarticular injection of ligament-derived stem/progenitor cells (LSPCs) on ACL reconstruction. The results suggest that this scaffold combined with intraarticular injection of LSPCs promotes tendon and bone tunnel fusion during ACL reconstruction. Additionally, this treatment strategy improved cartilage degeneration and reduced joint fibrosis severity. Therefore, the combination of LSPCs injection and SDF-1-releasing silk scaffolds has been clinically proven to be an excellent strategy for ACL reconstruction.

A variety of growth factor gene modification techniques have been used to promote tendon-bone healing in recent years. The directed overexpression of cytokines technique can control the differentiation direction of stem cells and make them differentiate in a direction conducive to tendon-bone healing. Chen et al. (2016) evaluated the effect of bone morphogenetic protein 2 (BMP2) and basic fibroblast growth factor (bFGF) gene modified BMSCs on tendon-bone healing. This study suggests that bone marrow MSCs modified with the bFGF gene can promote healing after ACL reconstruction. In this process, the synergistic effect of BMP2 and bFGF was greater than that of single genes. Recently, Kang et al. (2022) studied the effect of RUNt-related transcription factor 1 (RUNX1) overexpressed BMSCs on stenon-bone healing after ACL reconstruction. The results suggest that the increased expression of RUNX1 can improve the ability of BMSCs to promote tendon-bone healing after ACL reconstruction. RUNX1 upregulated BMSCs contribute to tendon-bone healing, which is characterized by improved biomechanical strength at the tendon-bone interface and improved osteogenesis. The above study indicated that the gene expression of BMSCs was modified by genetic engineering to differentiate in a direction conducive to native tendon-bone healing, which provided a promising strategy for future ACL reconstruction research.

It has been found that MSCs perform their biological functions such as promoting bone formation by secreting exosomes (Wu et al., 2022a; Xu et al., 2022a; Han et al., 2022; Li et al., 2022). Therefore, mesenchymal stem cells-derived exosomes (MSC-Exos) have been widely investigated as a promising strategy for tissue regeneration. Li et al. (2022) explored the efficacy of MSC-Exos in promoting tendon-bone healing after ACL reconstruction. The effect of MSC-Exos on tendon-bone healing and its possible mechanism were investigated *in vitro* and *in vivo*. It has been confirmed that miR-23a-3p in MSC-Exos promotes tendon-bone healing after ACL reconstruction by targeting the inhibition of IRF1 and NF-κB pathways in macrophages, thereby promoting the polarization of M2 macrophages and reducing the inflammatory response at the tendon-bone interface. This study provides a theoretical basis for using exosomes to promote early tendon-bone healing. Similarly, Xu et al. (2022a) explored the effects of MSC-Exos derived from the subpatellar fat pad (IPFP) (IPFP-MSC-Exos) on tendon-bone healing and intraarticular graft growing in ACL reconstruction rat models. The results also suggest that IPFP-MSC-Exos accelerates tendon-bone healing and intraarticular graft reconstruction after ACL reconstruction by participating in immune regulation of macrophage polarization. Recently, Wu et al. (2022a) developed a new type of bone mesenchymal stem cell-derived exosomes (ION- Exos) driven by magnetic (iron oxide nanoparticle magnetic field, ION) to investigate whether ION- Exos play a more significant role than normal MSCs derived exosomes (MSC-Exos) in tenoon-bone healing. The results showed that IONP-Exos significantly prevented bone loss around the bone tunnel, promoted more bone inward growth in the tendon graft, increased fibrochondrogenesis at the tendon-bone tunnel interface, and induced a higher maximum failure load than MSC-Exos. Future tissue engineering will be able to benefit from this study’s new strategy for promoting tendon-bone healing.

### 3.3 Cytokines

It has been shown that vascular and bone formation are the main determinants of tendon-bone healing. The formation of blood vessels and bone requires a large number of cytokines, so the lack of cytokines will lead to poor healing (Figueroa et al., 2015). Researchers are trying to use a variety of cytokines to accelerate the tendon-bone healing process and enhance the quality of healing. At present, the main cytokines commonly used to promote tendon-bone healing include BMP-2, acidic FGF (aFGF), milk fat globulin E8 (MFG-E8), secreted leukocyte protease inhibitor (SLPI), etc (Table 4). In addition, PRP, as a source of abundant cytokines, has also been applied in the study of promoting tendon-bone healing (Zhang et al., 2019; Uno et al., 2022).

**TABLE 4:** Preclinical studies of cytokines in promoting tendon-bone healing in ACL reconstruction.

Intervening measure	Animal model	Efficacy in promoting tendon-bone healing	References
PRP	Rabbits	PRP promotes tendon-bone healing by releasing cytokines.	Ağır et al. (2017)
PRP Combined with Gelatin Sponge (GS)	Rabbit	PRP-GS can optimize PRP release and promote early healing of the tendon-bone junction more effectively.	Zhang et al. (2019)
Platelet-rich gel (PRG) + deproteinized bone (DPB) compound	Rabbit	PRG + DPB compounds can improve the tensile strength of the healing interface and reduces the enlargement of the bone tunnel.	Zhai et al. (2013)
BMP-2	Rabbit	BMP-2 induces bone formation by regulating the recruitment and differentiation of bone progenitor cells.	Kim et al. (2014)
aFGF delivered in collagen (aFGF/collagen)	Rabbit	aFGF/collagen can promote early healing of the tendon-bone interface.	Lu et al. (2018a)
Fibrin clots loaded with bFGF and CaPP	Rabbit	Fibrin clots loaded with bFGF and CaPP enhanced cell migration and proliferation, increased bioactivity at the tendon-bone interface, and promoted tendon-bone healing.	Zhang et al. (2016)
BMP-2 and PRF	Rat	The combination therapy effectively increases the levels of growth factors that contribute to angiogenesis and eases the inflammatory response at the site of injury.	Han et al. (2019b)
Collagen sponge (CS) as a delivery device for osteoprotegerin (OPG)/BMP-2	Rabbit	The combination can ensure the slow and stale release of OPG/BMP-2 and significantly promote the tendon-bone healing in rabbit ACL model.	Wei et al. (2020)
MFG-E8	Rat	MFG-E8 attenuates the inflammatory response by enhancing macrophage secretion and M2 polarization, which ultimately leads to reduced inflammatory bone loss, increased new bone formation around the tunnel, and improved bone integration.	Geng et al. (2022)
SLPI	Rat	SLPI can effectively promote early tendon - bone healing after ACL reconstruction by enhancing migration and osteogenic differentiation of bone marrow MSCs.	Wu et al. (2022b)

BMP-2 is a widely distributed glycoprotein in bone matrix, and its main function is to promote osteoblast differentiation and induce bone formation (Durham et al., 2018). Many researchers have attempted to study the application of BMP-2 to promote tendon-bone healing. Kim et al. (2014) studied and evaluated the influence of BMP-2 insertion into bone tunnel on bone formation and tendon-bone healing. This study have demonstrated that BMP-2 local administration can promote bone formation in the bone tunnel, thereby improving the biomechanical strength of the tendon-bone junction and promoting tendon-bone healing. In order to improve the persistence of BMP-2 local application, researchers began to try to construct the drug delivery system of BMP-2. Han et al. (2019b) explored the effect of BMP-2 combined with platelet rich fibrin (PRF) on promoting tendon-bone healing. The study found that the systemic treatment effectively increased the levels of growth factors conducive to angiogenesis and alleviated the inflammatory response at the injured site. Similarly, Wei et al. (2020) prepared collagen sponge (CS) as a delivery device and supporting substrate for OPG/BMP-2, and then evaluated its effect on tendon-bone healing in rabbits. The results of this study suggest that the OPG/BMP-2/CS combination promotes fibrochondrogenesis and tendon-bone healing after ACL reconstruction. The above studies indicate that BMP-2, as a necessary cytokine for osteoblast differentiation and osteogenesis, has potential clinical application value in promoting tendon-bone healing, and improving its release pathway may achieve greater results.

Acidic fibroblast growth factor (aFGF), a member of the FGF family, significantly promotes osteoblast and chondrocyte mitosis and stimulates the formation of new capillaries (de Girolamo et al., 2015). Moreover, aFGF promotes not only proliferation and maturation of chondrocytes, but also migration and colony formation (Belov and Mohammadi, 2013). An evaluation of aFGF/collagen growth factor’s effect on rabbit tendon-bone interface healing was conducted (Lu et al., 2018a). It was found that the application of aFGF/collagen composite can promote early healing of the tendon-bone interface, especially with high concentrations of aFGF. Furthermore, Zhang et al. (2016) studied the effect of fibrin clots loaded with bFGF and ceramide-activated protein phosphatase (CaPP) on promoting tendine-bone healing. *In vivo* and *in vitro* studies, fibrin clots loaded with bFGF and CaPP were found to enhance cell migration and proliferation and expression of related genes and proteins, which increased biological activity at the tendon-bone interface and led to histological improvements in tendon-bone healing. These studies suggest that aFGF combined with other therapeutic measures can be used to promote tendine-bone healing. As shown above, aFGF is also a potential cytokine for promoting tendon-bone healing that can be applied clinically.

MFG-E8 is widely present in various mammalian cells and tissues, which can stimulate macrophages to develop into anti-inflammatory M2 phenotype and participate in immune regulation during the inflammatory process of the body (Gao et al., 2021). Therefore, it is involved in the angiogenesis of skin wounds (Uchiyama et al., 2014), as well as the healing of tendon rupture and other physiological processes (Shi et al., 2019). Geng et al. (2022) found in an animal model of ACL reconstruction that MFG-E8 attenuates the inflammatory response by enhancing macrophage secretion and M2 polarization, which ultimately leads to reduced inflammatory bone loss, increased new bone formation around the tunnel, and improved bone integration. Therefore, MFG-E8 can also be used as a novel therapeutic strategy to promote tendon-bone healing in ACL reconstruction patients. In addition, SLPI is a serine protease inhibitor, belonging to the whey acid protein family, which is commonly expressed and secreted in macrophages, epithelial cells and neutrophils (Antoniades et al., 2014). SLPI’s anti-inflammatory, antibacterial, and growth-regulating effects have made it highly studied in numerous fields, including wound repair, infection prevention, and cell proliferation (Wagenblast et al., 2015; Vigo et al., 2019). Recently, Wu et al. (2022b) verified the efficacy of SLPI in promoting tendon-bone healing. By enhancing migration and osteogenic differentiation of BMSCs, SLPI can effectively promote early stenon-bone healing.

It is well known that PRP, as a source of many cytokines, has been widely used in tissue repair and wound healing. PRP has also been tested to promote bone and tendon healing in recent years. Ağır et al. (2017) studied the effect of PRP on bone and tendon healing for ACL reconstruction. Results indicate that the use of PRP during sinusobone implantation is beneficial histologically. However, no significant effect was found to promote tendon-bone healing. The above results may be due to the fact that PRP alone in local application may not achieve sustained release. Zhang et al. (2019) explored the promoting effect of PRP combined with gelatin sponge (GS) on the healing and structure formation of the tendon-bone interface. *In vitro* GS-supported PRP enhanced PRP bioactivity and promoted MSCs proliferation and osteogenic gene expression. It promoted early healing of the tendon-bone junction in the rabbit model of ACL reconstruction. Similarly, Zhai et al. (2013) studied the effect of platelet-rich gel (PRG) + deproteinized bone (DPB) complex on tendon-bone healing. It was found that PRG + DPB compound could effectively trigger tendon-bone healing by promoting the maturation and ossification of tendon bone tissue. As a result, the healing interface is reinforced and the bone tunnel’s enlargement is reduced. The above studies indicate that PRP has great potential in promoting tendon-bone healing, and its important function can only be played by selecting effective drug carriers.

### 3.4 Mechanical stimulation

Numerous studies have found that MSCs can enhance proliferation and differentiation and inhibit apoptosis under mechanical stimulation (Chen and Jacobs, 2013; Aisha et al., 2015; Bin et al., 2016). Moreover, mechanical stimulation can promote MSCs regenerative potential, improving bone healing (Song et al., 2017; Xu et al., 2022b). As non-invasive mechanical energy, extracorporeal shock wave (ESW) and low intensity pulsed ultrasound (LIPUS) have been shown to be safe and convenient adjoint therapy to promote recovery from musculoskeletal injuries. It has been widely used in preclinical studies to promote tendon-bone healing and has achieved good results (Table 5).

**TABLE 5:** Preclinical studies of mechanical stimulation in promoting tendon-bone healing in ACL reconstruction.

Intervening measure	Animal model	Efficacy in promoting tendon-bone healing	References
ESW	Rabbit	ESW upregulates the expression of fibrochondrochondroid-related manufacturers and cytokines by providing mechanical signals.	Chow et al. (2014)
LIPUS	Rabbit	LIPUS can promote the formation and remodeling of more fibrochondral layers at the tendon-bone interface.	Lu et al. (2016a)
LIPUS	Rabbit	LIPUS therapy can accelerate bone formation during the healing process of the tendon-bone junction and significantly improve the healing quality of BTJ injuries.	Lu et al. (2016b)
LIPUS	Rabbit	The healing effect of LIPUS stimulation twice a day is better than that of the treatment once a day	Lu et al. (2018b)

ESW treatment was found to induce the migration of MSCs from other tissues and the flow of chondrocytes to enhance fibrochondral regeneration during tendon-bone healing (Kuo et al., 2007; Greve et al., 2009). Chow et al. (2014) explored the effect of ESW on promoting tendon-bone healing after CAL reconstruction, and found that ESW upregulated the expression of fibrochondroscope-related cytokines by providing mechanical signals. In a rabbit CAL reconstruction delayed repair model, a single ESW treatment enhanced stromal properties at the interface of fibrocartilage regeneration and healing. This study provides evidence for ESW as a therapeutic method to promote fibrochondral regeneration in knee joint wound healing, and also provides a new idea for promoting tendon-bone healing after CAL reconstruction.

LIPUS, as a non-invasive mechanical energy, has been shown to be a safe and convenient adjunctive therapy to promote recovery from musculoskeletal injuries (Waseem et al., 2010; Salem and Schmelz, 2014). LIPUS transmits high frequency acoustic pressure waves and mechanical stresses through the skin to biological tissues. It has been successfully shown to stimulate fracture healing and bone growth in animal models and clinical trials. Lu et al. (2016a) conducted a large number of studies using LIPUS to promote tendon-bone healing after CAL reconstruction. First, the study determined the optimal time for LIPUS treatment. The study found that the peripheral blood white blood cell count of animal models treated with LIPUS significantly increased from day 1 to day 3 after surgery, and there was more formation and remodeling of fibrocartilage layer at the tendon-bone interface, as well as more new bone formation. Secondly, they also investigated the effect of post-inflammatory LIPUS therapy on the healing process of bone-tendon junction in rabbits (Lu et al., 2016b). The results of the study showed that the area and length of new bone in the LIPUS group were significantly greater than that in the control group at the 8th and 16th week after surgery, and the formation and reconstruction of new bone was earlier than that in the control group. This study demonstrated that LIPUS therapy can accelerate bone formation during the healing process of the tendon-bone junction and significantly improve the healing quality of CAL injury. Finally, they evaluated the dose-effect of low-intensity pulsed ultrasound stimulation on tendon-bone healing. The study found that twice a day of low-intensity pulsed ultrasound stimulation was more effective than once a day therapy in promoting tendon-bone healing (Lu et al., 2018b). The above studies indicate that LIPUS is effective in promoting tendon-bone healing after CAL reconstruction. Early postoperative use and a frequency of 2 times a day may provide the greatest benefit to the patient. More clinical studies are needed to confirm the above theory to promote the wide clinical application of LIPUS.

## 4 Clinical study of strategies to promote tendon-bone healing in ACL reconstruction

A number of strategies for promoting tendon-bone healing have been shown in preclinical studies to have potential clinical applications. Some of the techniques have also been used in clinical studies (Table 6), where they have shown significant efficacy. However, due to the small number of clinical studies on the above strategies, there is still no breakthrough in clinical application at present.

**TABLE 6:** Clinical studies on promoting tendon-bone healing in ACL reconstruction.

Intervening measure	Patients (number)	Treatment outcomes	References
ADRCs	20	ADRC can significantly improve patient knee function and graft healing/maturation.	Alentorn-Geli et al. (2019)
CaP hybridization method for graft tendon	8	CaP hybridization is a safe and feasible method for ACL reconstruction. In addition, the method can improve clinical efficacy.	Mutsuzaki et al. (2019)
allogeneic hUCB-MSCs	10	Allogeneic hUCB-MSCs were safely used for ACL reconstruction without treatment-related adverse events. However, studies have not shown any evidence of clinical advantage.	Moon et al. (2021)
PRP	85	PRP can promote graft tendon-bone healing and improve early postoperative status and joint function.	Chen et al. (2022)
PRP	30	PRP had no significant effect on promoting tendon-bone healing and improving knee function. However, PRP may promote intraarticular graft maturation.	Gong et al. (2022)
ESWT	26	ESWT can improve the Lysholm subjective score and reduce long-term dilatation of the mid-tibial tunnel after surgery.	Wang et al. (2014)

The calcium phosphate (CaP) hybridization method of alternately soaking transplanted tendons has been shown to be effective in preclinical studies promoting tendon-bone healing. Mutsuzaki et al. (2019) transplanted quadriceps tendon with CaP hybridization to reconstruct ACL. The results indicate that the CaP hybridization method is safe and feasible for ACL reconstruction in clinical trials. In addition, it is preliminarily found that the method can improve the clinical efficacy. More clinical randomized controlled trials are needed in the future to verify the superiority of CaP hybridization in promoting tendon-bone healing in ACL reconstruction.

Preclinical studies suggest that MSCs may be the most promising strategy for promoting tendon-bone healing. However, the efficacy shown in clinical studies has not been sufficient to warrant widespread clinical use. Alentorn-Geli et al. (2019) used a retrospective case-control study to compare the healing and clinical outcomes of patients undergoing intraoperative ACL reconstruction with ADRCs and without ADRCs. The results of this study showed that patients who received ADRCs during ACL reconstruction had significantly improved knee function and graft maturity at 12 months. However, Moon et al. (2021) explored the clinical effect of allogeneic human cord blood-derived MSCs (hUCB-MSCs) on ACL reconstruction (KCT0000917). Allogeneic hUCB-MSCs were found to be safe for ACL reconstruction with no treatment-related adverse events at a 2-year follow-up. However, studies have not shown any evidence of clinical advantage. The differentiation directions of MSCs from different sources differ greatly, which may be the reason for the lack of good efficacy in the above studies. Therefore, more clinical studies are needed to confirm the clinical application of MSCs in promoting tendon-bone healing. In addition, gene modification to control the direction of MSCs differentiation should be more widely used in clinical studies to promote tendon-bone healing.

Preclinical studies of PRP in promoting tendon-bone healing have also demonstrated significant efficacy. However, current clinical studies have shown mixed results. Chen et al. (2022) used a retrospective case-control study to evaluate the effect of PRP on tendon-bone healing after ACL reconstruction. The results of the study showed that the PRP group Lysholm and International Knee Literature Committee score were significantly better than the control group at 3 months after surgery. None of the 85 patients in the PRP group had complications such as knee infection, vascular and nerve injury. In all patients, knee flexion was greater than 90°, straightness was 0°, and no joint stiffness was observed. This study suggests that PRP can promote graft muscle and bone healing and improve early postoperative knee function. However, Gong et al. (2022) studied the effect of PRP on tendon-bone healing and intraarticular graft maturation after ACL reconstruction (NCT04659447). The results showed that PRP had no significant effect on accelerating tendon-bone healing and improving knee function. It was found that PRP may promote the maturation of grafts. The mechanism by which PRP promotes tendon-bone healing is well established, and preclinical studies have also found that the duration of cytokine release during local application of PRP is correlated with the efficacy of promoting tendon-bone healing. Therefore, the combination of PRP with biological nanorostents or hydrogel microparticles may enhance its efficacy.

Mechanical stimulation can promote tendon-bone healing by inducing MSCs differentiation. Wang et al. (2014) evaluated the effects of extracorporeal shock wave therapy (ESWT) on human ACL reconstruction using a randomized controlled clinical study. In this study, 26 patients underwent autogenous hamstring tendon reconstruction and ESW immediately after surgery. Tibial tunnel dilatation was significantly reduced at 6 and 2 months in the ESWT group compared to the control group (*p* = 0.024 and *p* < 0.001). This study demonstrated that ESWT significantly improved the subjective Lysholm score after autologous ACL reconstruction and reduced long-term tibial tunnel enlargement after surgery. Therefore, ESWT as a non-invasive physiotherapy measure in promoting tendon-bone healing has certain value in clinical application.

## 5 Discussion and outlook

It is important to consider the risk factors that may affect tendon-bone healing during ACL reconstruction. Studies have shown that the effectiveness of tendon-bone healing after ACL reconstruction deteriorates with age (Magnitskaya et al., 2020; Best et al., 2021). In the treatment of this condition, a combination of strategies to promote tendon-bone healing may be considered to compensate for the loss of bone formation associated with aging. At present, interference screws are often used to fix the transplanted tendon. However, the application of interfering screws has been found to be associated with complications (Xu et al., 2021; Ammann et al., 2022). Therefore, for patients at risk of poor tendon-bone union, the fixation strategy needs to be optimized for optimal clinical benefit. Secondly, the flat shape of bone marrow canal and the manual drilling method during the establishment of bone tunnel are recommended for clinical promotion and application. After all, both physiological and physical perspectives suggest that these changes may lead to an optimal healing microenvironment for tendon-bone healing (Zhao et al., 2019; Tei et al., 2020). Finally, studies have shown that low-intensity mechanical loading early after surgery promotes tendon-bone healing (Song et al., 2017). Therefore, it is more beneficial for clinical rehabilitation to choose this time point to instruct patients to take moderate activities of affected limbs. The maximum optimization in the above aspects during ACL reconstruction is believed to significantly reduce the recurrence rate.

With the rapid development of bioengineering technology, the application of biological materials in the field of tissue repair has become increasingly hot. Strontium-rich calcium phosphate bone cement (Weimin et al., 2013; Patel et al., 2018), hydrogel microspheres (Oka et al., 2013; Huang et al., 2020), nano-biomimetic scaffolds (Han et al., 2015; Kim et al., 2015; Chou et al., 2016), hydroxyapatite (Jiang et al., 2023) and other bone inorganic components have been applied in the study of promoting tendon-bone healing. Preclinical studies have all achieved good curative effect, but the results of clinical studies have not achieved satisfactory results. On the one hand, it may be because there are few clinical studies at present, and some potential strategies have not been studied. On the other hand, due to the difficulties in the functional assessment of patients after ACL reconstruction, most studies are limited to animal models. The role of biomaterials in promoting tendon-bone healing is mainly to act as scaffolds of fibrocytes and osteoblasts in bone tunnels to promote bone formation (Bi et al., 2021; Lv et al., 2022; Han et al., 2023), and secondly to act as drug carriers to fix drugs in the knee joint for continuous and effective release within a certain period of time (Lee et al., 2017; Franklin et al., 2020; Huang et al., 2020), so as to achieve the optimal purpose of promoting tendon-bone healing. Therefore, the application of biomaterials should be widely combined with other therapeutic strategies, such as stem cells, cytokines, etc.

Preclinical studies have demonstrated that MSCs can promote tendon-bone healing and reduce the expansion of bone tunnels (Jang et al., 2015; Chen et al., 2021). Due to the differences in the differentiation directions of MSCs from different sources, the efficacy of different MSCs in promoting tendon-bone healing varies greatly (Kang et al., 2022). In order to overcome this problem, researchers tried to use gene modification technology to control the differentiation direction of MSCs, and achieved success in basic research (Chen et al., 2016; Kang et al., 2022). It solves the problem that MSCs from different sources can be applied to promote tendon-bone healing. In addition, studies have found that MSCs exercise their immunomodulatory biological functions by secreting exosomes, and researchers have attempted to use MSC-Exos to promote tendon-bone healing (Wu et al., 2022a; Xu et al., 2022a; Li et al., 2022), results in animal models also showed good efficacy. Therefore, the application of the above MSCs optimization strategies in clinical studies should be widely carried out to accelerate the clinical application of the above strategies.

As essential components of tissue injury healing, cytokines play an important role in promoting tendon-bone healing, which has been confirmed by preclinical studies (Tian et al., 2015; Han et al., 2019b; Wu et al., 2022b). In addition, PRP, as a source of rich cytokines, will be used to promote tendon-bone healing. Studies have found that the rapid release of cytokines and PRP strategies used alone cannot be fixed in the bone tunnel in a certain period of time (Ağır et al., 2017), which leads to the failure of cytokine release to cooperate with the physiological process of tendon-bone healing, so that cytokines cannot play their intended role. The application of cytokines and PRP should be conventional combined with hydrogel microspheres or nano-bionic scaffolders to achieve simultaneous cytokine release and tendon-bone healing.

Studies have shown that MSCs can enhance proliferation and differentiation under mechanical stimulation, and further promote tendon-bone healing (Song et al., 2017). By virtue of this mechanism, ESWT and LIPUS have been applied in the study of promoting tendon-bone healing. And in preclinical studies, good results have been shown. In clinical studies on ESWT, it has been found that immediate use of ESWT after ACL reconstruction can reduce the enlargement of bone marrow tract in the long term (Wang et al., 2014). Some of the strategies discussed above work to promote fibrochondral and bone formation early after surgery, and these strategies combined with mechanical stimulation may result in better outcomes. However, more clinical studies are needed to confirm this.

## 6 Conclusion

In conclusion, tendon-bone healing after ACL reconstruction determines the overall outcome of surgery. Age, fixation of tendon graft, establishment of bone tunnel, and mechanical load of tendon graft after operation are all risk factors that may affect tendon-bone healing. To further promote tendon-bone healing, various therapeutic strategies have been developed, including biomaterials, stem cell transplantation, cytokines, and mechanical stimulation. In clinical treatment, factors that may affect tendon-bone healing should be optimized based on individual conditions of each patient, and strategies to promote tendon-bone healing should be assisted if necessary. Only in this way can a breakthrough be made in promoting tendon-bone healing after ACL reconstruction.
